# Translatomics Probes Into the Role of Lycopene on Improving Hepatic Steatosis Induced by High-Fat Diet

**DOI:** 10.3389/fnut.2021.727785

**Published:** 2021-11-02

**Authors:** Tengda Huang, Jingsu Yu, Zeqiang Ma, Qinghua Fu, Siqi Liu, Zupeng Luo, Kang Liu, Lin Yu, Weiwei Miao, Dongling Yu, Ziyi Song, Yixing Li, Lei Zhou, Gaoxiao Xu

**Affiliations:** ^1^Key Laboratory of Embryo Development and Reproductive Regulation of Anhui Province, Fuyang Normal University, Fuyang, China; ^2^State Key Laboratory for Conservation and Utilization of Subtropical Agro-Bioresources, College of Animal Science and Technology, Guangxi University, Nanning, China; ^3^Teaching and Research Section of Biotechnology, Nanning University, Nanning, China

**Keywords:** translatomics, Ribo-seq, lycopene, high fat diet, NAFLD

## Abstract

Liver is an important organ for fat metabolism. Excessive intake of a high-fat/energy diet is a major cause of hepatic steatosis and its complications such as non-alcoholic fatty liver disease and non-alcoholic steatohepatitis. Supplementation with lycopene, a natural compound, is effective in lowering triglyceride levels in the liver, although the underlying mechanism at the translational level is unclear. In this study, mice were fed a high-fat diet (HFD) to induce hepatic steatosis and treated with or without lycopene. Translation omics and transcriptome sequencing were performed on the liver to explore the regulatory mechanism of lycopene in liver steatosis induced by HFD, and identify differentially expressed genes (DEGs). We identified 1,358 DEGs at the translational level. Through transcriptomics and translatomics joint analysis, we narrowed the range of functional genes to 112 DEGs and found that lycopene may affect lipid metabolism by regulating the expression of *LPIN1* at the transcriptional and translational levels. This study provides a powerful tool for translatome and transcriptome integration and a new strategy for the screening of candidate genes.

## Introduction

The liver is one of the vital metabolic organs and the main area of lipid metabolism. The liver not only utilizes lipids to produce energy, but also secretes lipids in the form of very low-density lipoproteins (VLDLs) (1). However, high-fat/energy diet can cause lipid deposits in the liver (2), which may increase the risk of non-alcoholic fatty liver disease (NAFLD). A recent epidemiological study has shown that 12–38% of adults are suffering from NAFLD (3). Due to the high incidence of NAFLD in the population, its hazard to health, and limited treatment options (4, 5), there is an urgent need to develop new drugs and therapeutic strategies.

Lycopene (ψ,ψ-carotene) is a polyene, mainly found in ripe tomatoes, watermelon, guava, rose hips, papaya and grapefruit (6, 7). During recent decades, studies on the function of lycopene have mainly focused on its antioxidative, lipid-lowering, anti-inflammatory and antitumor effects (8–11). Some research has shown that lycopene can inhibit hepatic steatosis by inducing downregulation of fatty acid binding protein 7 through regulating miRNA-21 (12). In another study, the lycopene metabolite, apo-10'-lycopenoic acid, protects against the development of steatosis in ob/ob mice by upregulating SIRT1 gene expression and activity (13). Metabolomics research has revealed that lycopene can increase the levels of metabolites related to the antioxidant response, to alleviate steatosis induced by high-fat diet (HFD) (14). The regulatory effect of lycopene on lipid metabolism has been reported in miRNA (12), RNA (13), and metabolomics (14) studies, but the lack of studies at the level of gene translation has limited our further understanding of the lipid-lowering effect of lycopene.

Proteins are deeply involved in all aspects of cellular, physiological and developmental processes, such as cell growth and division, organogenesis, and reproductivity (15–17). Therefore, researchers have focused on protein expression. However, proteomics has some shortcomings. The sensitivity of protein profile detection is low, and it is difficult to detect low-abundance proteins, which may lead to some proteins with important biological functions, but low expression not being detected (18). RNA sequencing (RNA-seq) has the advantages of high throughput, high sensitivity and low cost; therefore, scientists usually use RNA-seq to detect the abundance of gene transcriptional expression to evaluate the abundance of protein expression (19). However, in recent years, it has been recognized that there is a poor correlation between mRNA and protein abundance. Gygi et al. found that there were 20–30-fold differences in mRNA and protein expression levels after comparing 106 yeast proteins (20), which indicated that it is inappropriate to regard gene transcriptional abundance as protein abundance (21). From RNA to protein, there are many processes such as RNA degradation, splicing, non-coding RNA regulation, epigenetic modification, and protein processing. Translatomics can fill the long omics gap between RNA and protein quantification and facilitate the study of gene expression regulation more directly. Ribosome footprint sequencing (Ribo-seq) is a recently developed method that can measure translational activity in a genome-wide and quantitative manner by base pair resolution (22), which opens a new way to understand biological problems.

This study depicted the molecular portrait of mice liver with or without lycopene treatment from translatome, which fills the gap in the research on the translation level of the lipid-lowering effect of lycopene. The purpose of the present study was to explain the regulatory mechanism of lycopene in liver lipid metabolism and identify differently expressed genes (DEGs) through the joint analysis of translatomics and transcriptomics. The results can provide a method for screening candidate genes and further understanding the mechanism of lycopene for the treatment of NAFLD.

## Materials and Methods

### Animals and Feeds

The C57/BL6 mice used in the experiment were purchased from Guangxi Medical University. Mice were randomly divided into three groups (*n* = 8 per group) and fed with different diets from week 8: CK: standard feed (10% kcal, Supplementary Table 1), HFD: high-fat feed (45% kcal, Supplementary Table 1), and LYC: high-fat feed containing lycopene (0.33% w/w) (23, 24). The feed for mice was purchased from Jiangsu Medicience Biopharmaceutical Co. Ltd., and the lycopene mixed into the feed was purchased from Xian Baichuan Biotechnology Co. Ltd. Animals were housed in a pathogen-free barrier environment, on a 12/12-h dark/light cycle throughout the study, and were supplied water *ad libitum*. After 8 weeks on different diets, the mice were euthanized.

### Oil Red O Staining

Liver tissue was prepared in frozen sections, which were stained with Oil Red O solution in 60% isopropanol for 10 min, and then counter-stained with hematoxylin for 1 min. The slides were viewed at 200 × magnification.

### Cell Culture

HepG2 cells were purchased from the Cell Bank of the Chinese Academy of Sciences Shanghai Institute of Cell Biology (Shanghai, China) and maintained in Dulbecco's modified Eagle's medium (DMEM) supplemented with 10% fetal bovine serum under an atmosphere of 5% CO_2_ at 37°C. At 80% confluence, the DMEM was changed to DMEM supplemented with oleic (200 μM) and palmitic (100 μM) acids (OAPA), simulating a high-fat environment. Lycopene (98%) was purchased from the China Beijing Solarbio Science & Technology Co., Ltd., dissolved in tetrahydrofuran containing 250 ppm butylated hydroxytoluene (99%; Shanghai Industrial Co., Ltd., Shanghai, China) and diluted to 10 μ M with fetal bovine serum. The cells were collected after 24 h of incubation.

### Triglyceride (TG) Measurement

TG content of the cells and tissues were measured using the TG assay kit (Pulilai, Beijing, China), as described previously (25).

### RNA Extraction and Quantitative Polymerase Chain Reaction (qPCR)

Total RNA from tissues and cells was extracted using TRIzol reagent and cDNA was synthesized using PCR conditions of 95°C for 3 min, followed by 40 cycles of 95°C for 10 s, 60°C for 1 min, and 72°C for 10 s. Gene expression levels were measured by quantitative PCR using the 2^−ΔΔCt^ method with β*-actin* as an internal control. The forward and reverse primers were as follows: *LPIN1*: TAAACGGAGCCGACACCTTGGA and CCGTTGTCACTGGCTTGTTTGG; β*-actin*: AACAGTCCGCCTAGAAGCAC and CGTTGACATCCGTAAAGACC.

### Western Blot

Tissue was lysed in RIPA lysis buffer (Solarbio, Beijing, China) containing 1 mM PMSF. The total protein concentration was determined using a BCA protein assay kit (Beyotime, Shanghai, China). The centrifuged supernatant was boiled and SDS-PAGE electrophoresis was performed (Mini-PROTEAN Tetra System, Bio-Rad), followed by transfer to a PVDF membrane. Subsequently, the primary antibodies anti-LPIN1 rabbit polyclonal antibody (1:1,000; D163631, Sangon Biotech, Shanghai, China), β-tubulin antibody (1:1,000; 2146s, Cell Signaling Technology, Inc., Shanghai, China) were incubated overnight at 4°C. The Image Lab (Universal Hood II, Bio-Rad) was used to detect chemiluminescent signals after the secondary antibody incubation.

### Ribo-seq

Total ribosome footprints (RFPs) extraction from liver tissue of mice was performed as previously described (26). Liver was pre-treated with 100 mg/ml cycloheximide for 15 min, washed with pre-chilled phosphate-buffered saline, then treated with cell lysis buffer {1% Triton X-100 in ribosome buffer [RB buffer, 20 mM HEPES–KOH (pH 7.4), 15 mM MgCI_2_, 200 mM KCl, 100 μg/ml cycloheximide and 2 mM dithiothreitol]}. Cell debris was removed by centrifugation at 16,200 × g for 10 min at 4°C. Supernatants were transferred into new pre-chilled 1.5-ml tubes with the addition of 2 μl Ribolock RNase Inhibitor (40 U/μl, Fermentas) in each tube. RNase I (10 U/μl, Fermentas) was added at 0.2 μl per tube, followed by incubation at 37°C for 15 min and reaction termination with 1% sodium dodecyl sulfate (1/10 volume per tube). The digested samples were pooled and layered on the surface of 15 ml sucrose buffer (30% sucrose in RB buffer). The ribosomes were pelleted by ultracentrifugation at 185,000 × g for 5 h at 4°C. Ribosome protected fragment (RPF) extraction was performed using the TRIzol method and rRNA was depleted using the Ribo-Zero rRNA Removal Kit (Mouse) (Epicenter).

### RNA-seq

Total RNA was isolated using TRIzol reagent. Equal amounts of total RNA or RFPs from the HFD and LYC groups were prepared for subsequent library construction and high-throughput sequencing. RNA libraries were prepared according to the protocol of the VAHTS mRNA-seq v.3 Library Prep Kit for Illumina (Vazyme Biotech Co. Ltd., Nanjing, Jiangsu, China), and the raw sequencing reads were generated on an Illumina HiSeqX Ten sequencer.

### Sequence Analysis

We used cutadapt and bowtie2 software to process raw data to obtain high-quality clean data. The clean data were compared to the reference genome (GRCm38/mm10) using STAR software. The FANSE2 series algorithm (27, 28) was used for quantitative genetic analysis. The mRNA and RPFs in each sample were normalized using reads per kilobase per million reads (RPKM) (29). Differential gene calculation was carried out for the identified genes by edgeR (30) software, and the screening threshold was |log2 fold change|>1 and *p* < 0.001. Gene Ontology (GO) and Kyoto Encyclopedia of Genes and Genomes (KEGG) analysis were performed using the OmicShare tools; a free online platform for data analysis (http://www.omicshare.com/tools).

### Statistical Analysis

The results were analyzed using GraphPad Prism 8 and presented as the mean ± standard error of the mean. The student's *t*-test was used to determine the significance of the difference between two groups, and the differences were considered statistically significant if *p* < 0.05.

## Results

### Lycopene Alleviates TG Deposition Induced by High-Fat Diet

**Figure 1:**
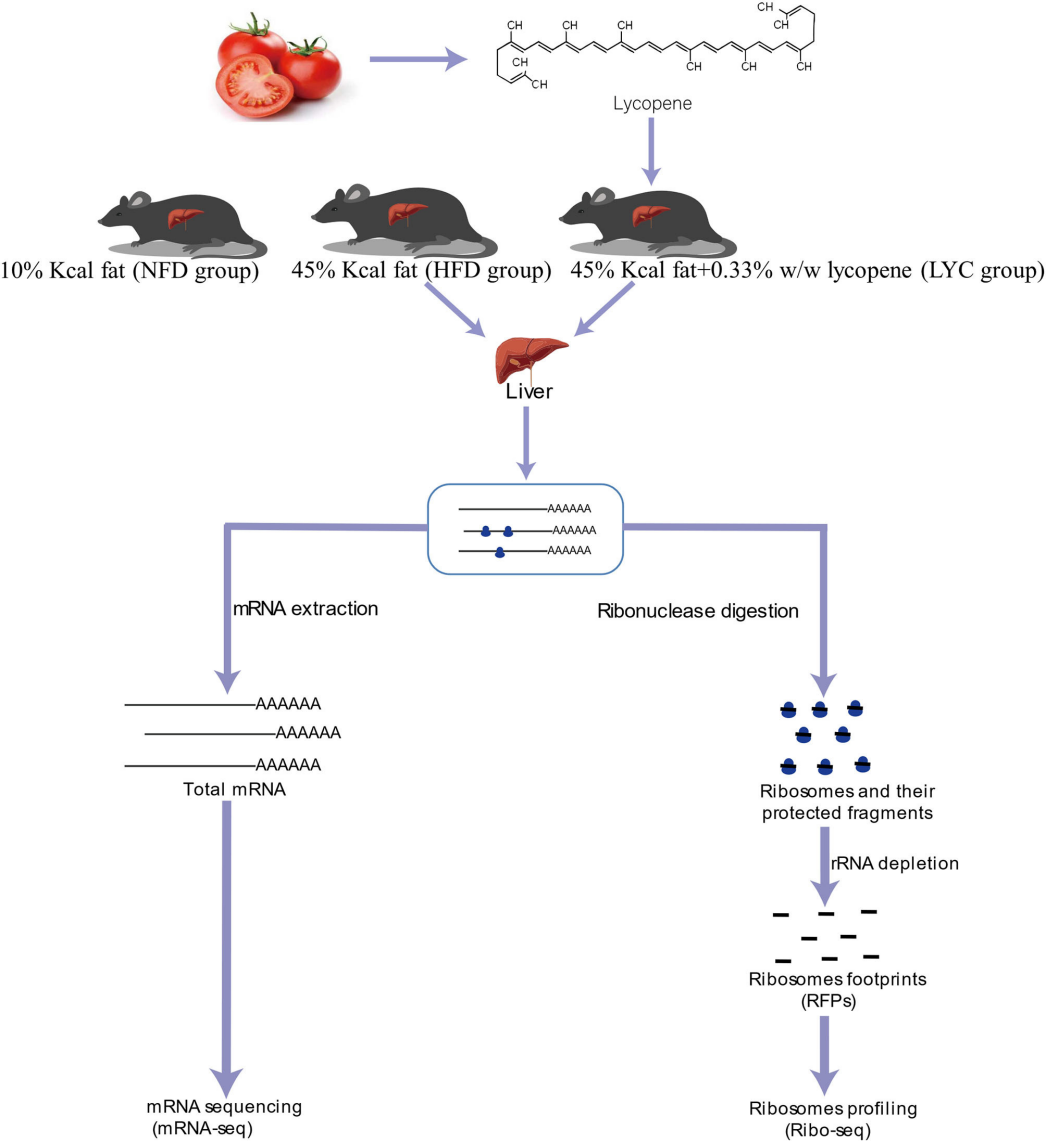
Schematic of the study design and high-throughput sequencing.

**Figure 2:**
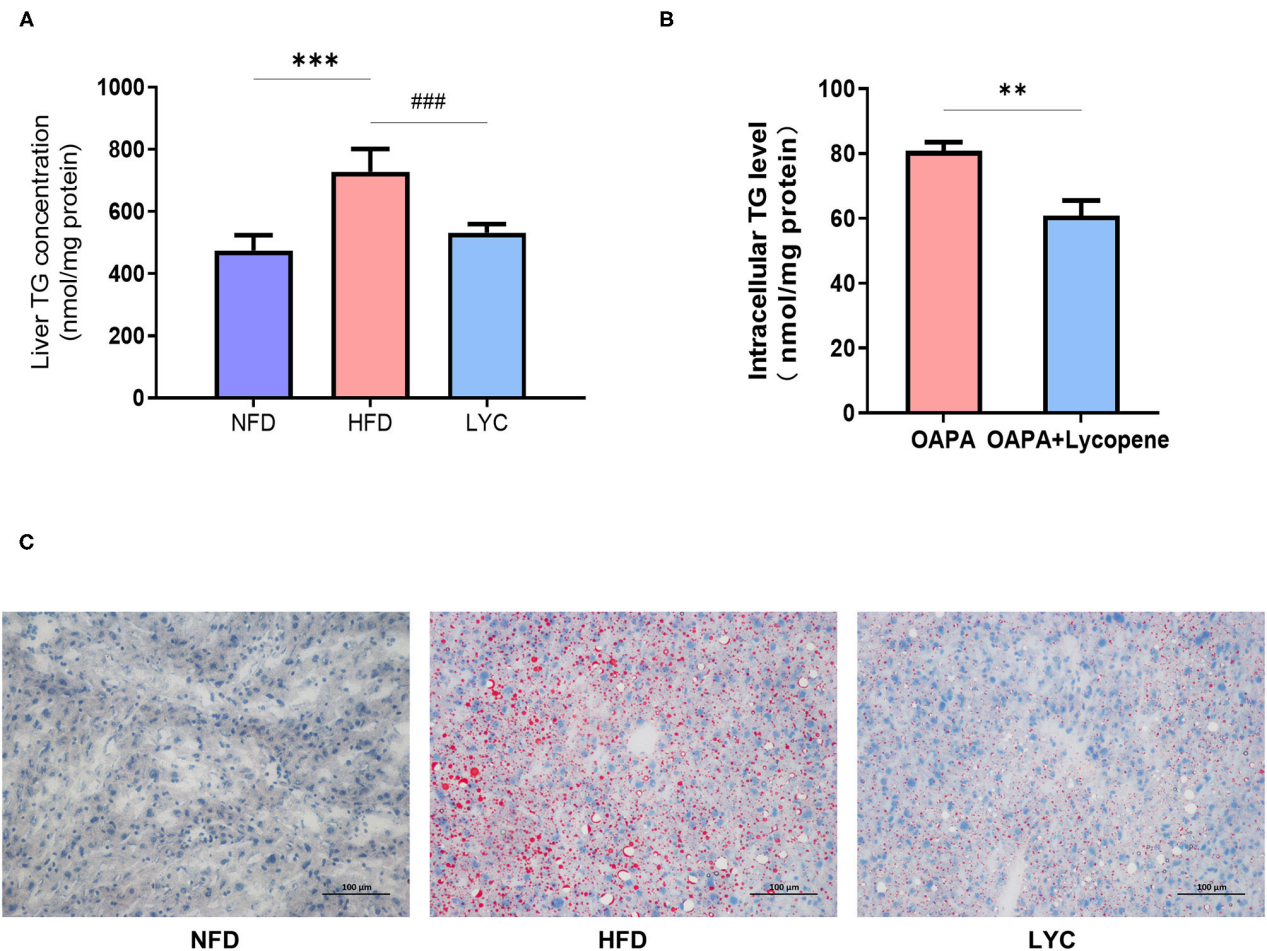
Lycopene alleviates liver fatty deposition induced by HFD. **(A)** Mouse liver TG level. **(B)** Oil Red O staining (×200 magnification) of the liver in different groups. Scale bar = 100 μm. **(C)** HepG2 cell TG level. Data are expressed as the mean ± SD. ^**^p< 0.01, ^***^p or ^###^ *p* < 0.001. NFD, normal fat diet group; HFD, high fat diet group; LYC: lycopene group.

Figure 1 shows the schematic of the study design and high-throughput sequencing. After 8 weeks of feeding, the liver TG level under HFD was significantly increased (*p* < 0.001, Figure 2A), but the liver TG level of mice fed HFD supplemented with lycopene (LYC) was significantly lower than in those fed HFD alone (*p* < 0.001, Figure 2A). We also studied the effect of lycopene on lipid metabolism in HepG2 cells by adding OA and PA to DMEM to simulate a high lipid environment. The results showed a 25% decrease in intracellular TG level in the OAPA + lycopene group compared to the OAPA group (*p* < 0.01, Figure 2B). Oil Red O staining showed that a large number of lipid droplets accumulated in the liver of the HFD group, while the number of lipid droplets was significantly reduced in the LYC group (Figure 2C).

### Overview of Ribo-seq Results

**Figure 3:**
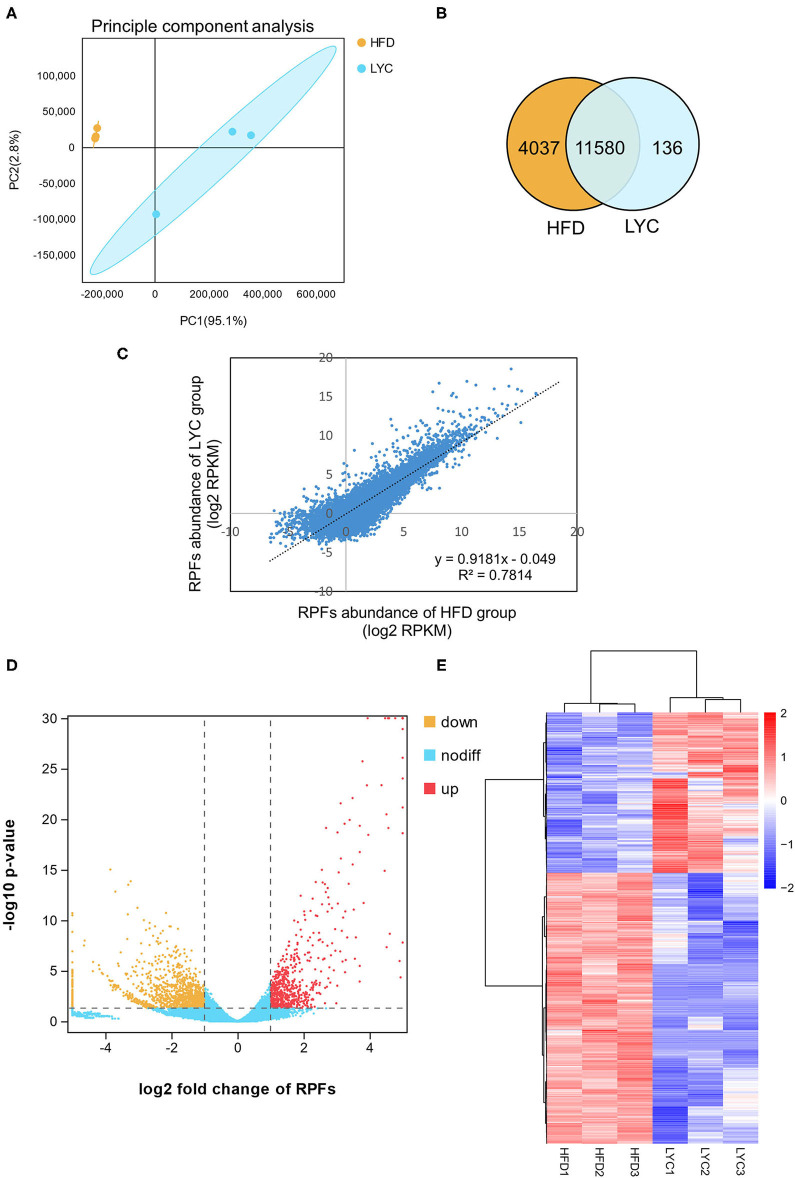
Overview of Ribo-seq results in HFD and LYC mice livers. **(A)** Principal component analysis of Ribo-seq. **(B)** Venn diagram showing number of common genes among the two groups. **(C)** RPFs abundance correlation scatter plot between HFD and LYC livers. **(D)** Volcano plots indicate the directionality of significant DEGs. Genes upregulated (red) or downregulated (orange) by supplement lycopene correspond to a 1.0 decrease or increase in log_2_ fold change with *p* < 0.01. **(E)** A total of 1,358 DEGs were clearly distinguished based on their Ribo-seq abundance. The color key (from blue to red) of *Z*-score value (−2–2) indicated low to high expression levels. HFD1, HFD2, and HFD3 represent group HFD biological repetition 1, 2, and 3. LYC1, LYC2, and LYC3 represent group LYC biological repetition 1, 2, and 3.

To investigate the underlying mechanism of lycopene on improving hepatic steatosis, we used a translatome analysis to identify the liver DEGs between the HFD and LYC groups. Six cDNA libraries, including three biological replicates from the HFD group (HFD1, HFD2, and HFD3) and three biological replicates from the LYC group (LYC1, LYC2, and LYC3) were constructed and analyzed by high-throughput sequencing. The principal component analysis of six samples was calculated and showed high-level repeatability of intraclass samples (Figure 3A). The overlapping genes in the HFD and LYC groups were counted, and there were 11,580 genes detected in both groups (Figure 3B). The abundance of gene in the HFD and LYC groups had a high correlation (*R*^2^ = 0.7814, Figure 3C), which indicated that these two groups can be analyzed together. Subsequently, 1,358 DEGs of Ribo-seq were identified by |log_2_(fold change) |>1, *p* < 0.01, among which 505 were upregulated and 853 downregulated (Figure 3D and Supplementary Table 2). The 1,358 DEGs of Ribo-seq were demonstrated in a heat map based on gene expression levels (Figure 3E).

### GO and KEGG Functional Enrichment Analysis

**Figure 4:**
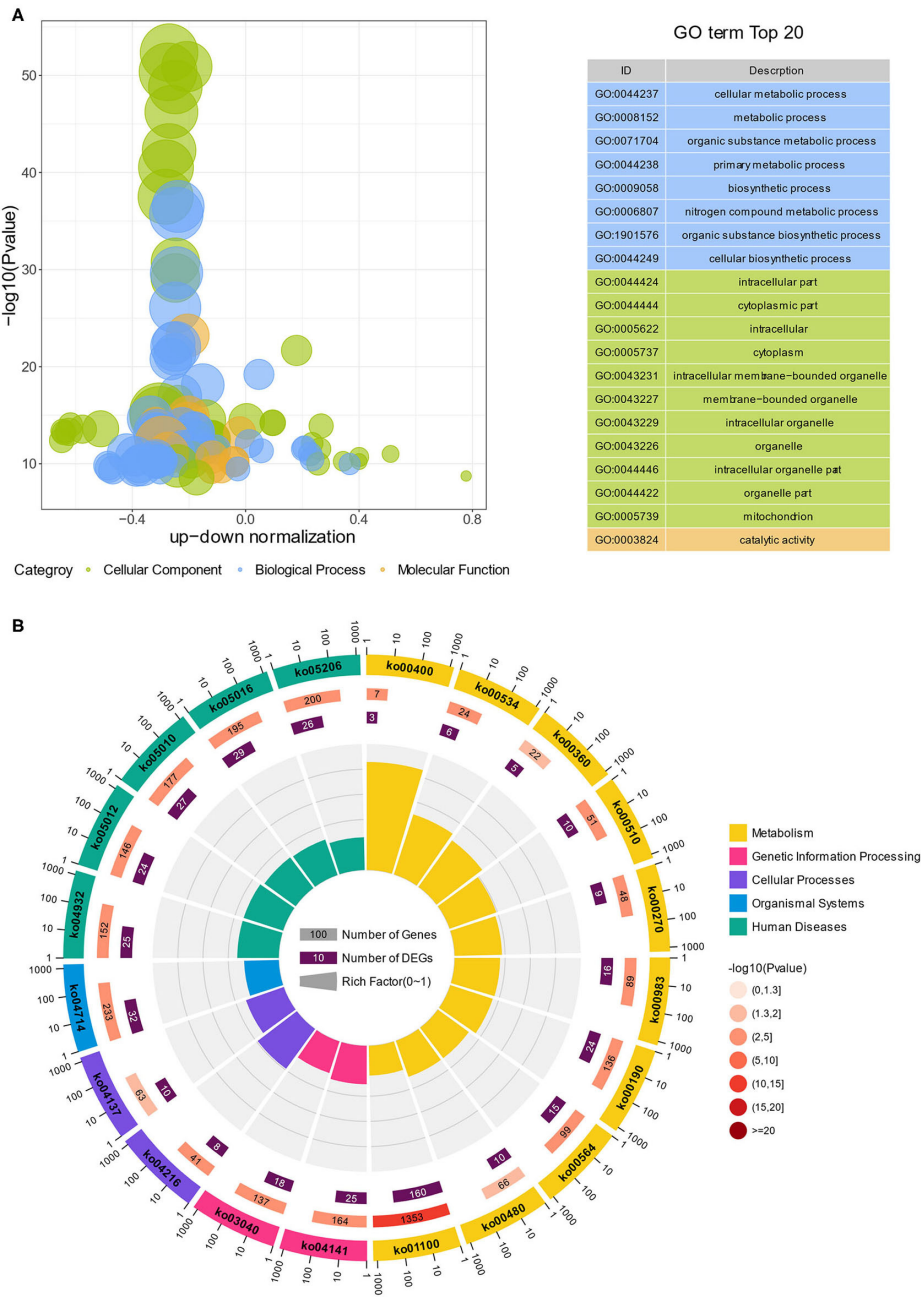
GO and KEGG analysis of DEGs in Ribo-seq. **(A)** GO bubble diagram showing the top 20 enriched GO terms. Three colors represent three GO categories, including cellular component, biological process and molecular function. **(B)** The enrichment circle diagram shows the KEGG analysis of the top 20 pathways. Four circles from the outside to the inside. First circle: the classification of enrichment, outside the circle is the scale of the number of genes. Different colors represent different categories. Second circle: number and *p*-values of the classification in the background genes. The more genes, the longer the bars, the smaller the value, the redder the color. Third circle: bar chart of the total number of DEGs. Fourth circle: rich factor value of each classification (number of DEGs in this classification divided by the number of background genes). Each cell of the background helper line represents 0.1.

GO and KEGG analyses were conducted to examine the functional pathways for DEGs. The top 20 GO terms, classified by –log_10_(*p-*value), were significantly enriched in DEGs of Ribo-seq compared to the genome background GO. The top 20 GO terms included eight biological process (BP), 11 cellular component (CC) and one molecular function (MF). The BP terms were enriched in cellular metabolic process, metabolic process, organic substance metabolic process (Figure 4A, Supplementary Table 3). The functional enrichment cycle diagram displayed the top 20 KEGG pathways, classified by –log_10_(*p*-value), which revealed that the DEGs of Ribo-seq were mainly enriched in five KEGG A classes, including metabolism, genetic information processing, cellular processes, organismal systems and human diseases. Among these pathways, metabolism included oxidative phosphorylation (ko00190) and glycerophospholipid metabolism (ko00564); genetic information processing included protein processing in endoplasmic reticulum (ko04141) and spliceosome (ko03040); cellular processes included ferroptosis (ko04216); organismal systems included thermogenesis (ko04714); human diseases included NAFLD (ko04932) (Figure 4B, Supplementary Table 4). As shown in Supplementary Figures 1A,B, the KEGG network diagram, respectively, showed 24 DEGs of Ribo-seq enriched in the oxidative phosphorylation pathway and 25 DEGs of Ribo-seq enriched in the NAFLD pathway. In conclusion, our data indicated that lycopene can regulate lipid metabolism by effecting translational level, thus alleviating NAFLD.

### Lycopene Altered Gene Expression at Transcriptional and Translational Levels

**Figure 5:**
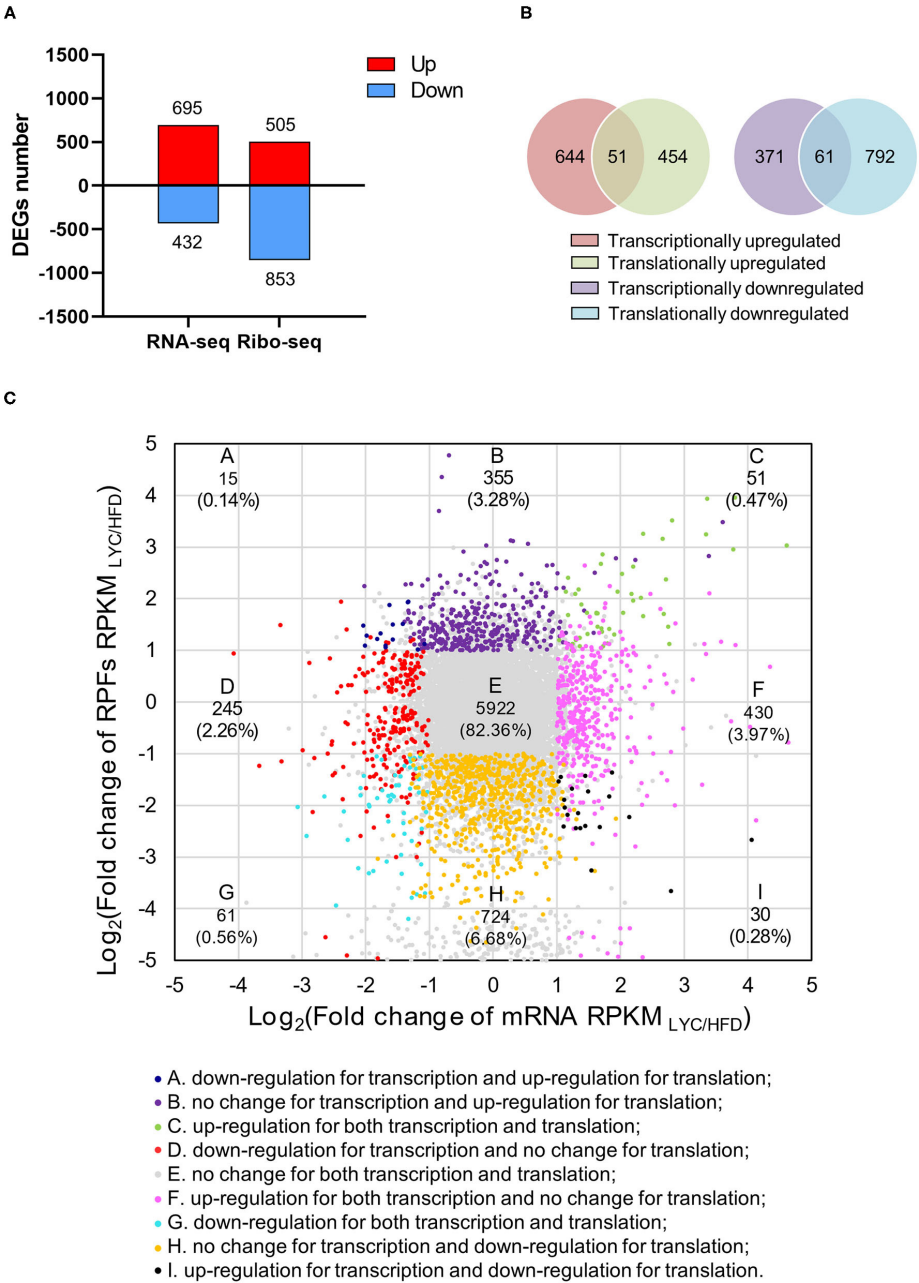
Lycopene altered gene expression at both transcriptional and translational levels. **(A)** Number of DEGs (|log_2_ fold change| ≥1 and *p* < 0.01) at transcriptional or translational levels. The red and blue bars refer to the number of upregulated and downregulated genes, respectively. **(B)** Venn diagram showing the relationship between transcriptome and translatome. **(C)** Fold changes of RPKMs at transcriptional and translational levels. Nine squares in different colors indicate nine responsive groups (|log_2_fold change| ≥1 and *p* < 0.01).

To further investigate the role of lycopene in regulating hepatic steatosis induced by HFD, we performed RNA-seq using the liver of mice in the HFD and LYC groups. In the RNA-seq data, there was a good correlation of biological duplications within the two groups (Supplementary Figure 2). The volcano map shows 1,127 DEGs in RNA-seq (Supplementary Figure 3 and Supplementary Table 5). Due to the high correlation of gene expression abundance between transcriptome and translatome, the two omics can be combined for analysis (*R*^2^ = 0.7225 and 0.5372, respectively; Supplementary Figure 4). We detected 695 upregulated and 432 downregulated genes at the transcriptional level, and 505 upregulated and 853 downregulated genes at the translational level (Figure 5A). Only 51 DEGs were upregulated in both the transcriptome and translatome, and only 61 DEGs were downregulated in both the transcriptome and translatome (Figure 5B). Based on the fold change of RPKM (| log_2_(fold change) | > 1 and *p* < 0.01 as cutoff), genes were classified into nine categories (Figure 5C and Supplementary Table 6). Further analysis revealed that 1.03% of the responsive genes (112 genes) were among the accordant groups (quadrant C and G), which were co-regulated with their expression increased or decreased to a similar extent at the transcriptional and translational levels. Meanwhile, 16.61% of the responsive genes (1,799 genes) were located in the other six discordantly regulated groups (classes A, B, D, F, H, and I; Figure 5C). By combining RNA-seq and Ribo-seq, we were able to narrow our focus to the 112 DEGs (C and G quadrants) that are regulated in the same direction as transcription and translation.

### Functional Enrichment Analysis of Protein–Protein Interaction Networks

**Figure 6:**
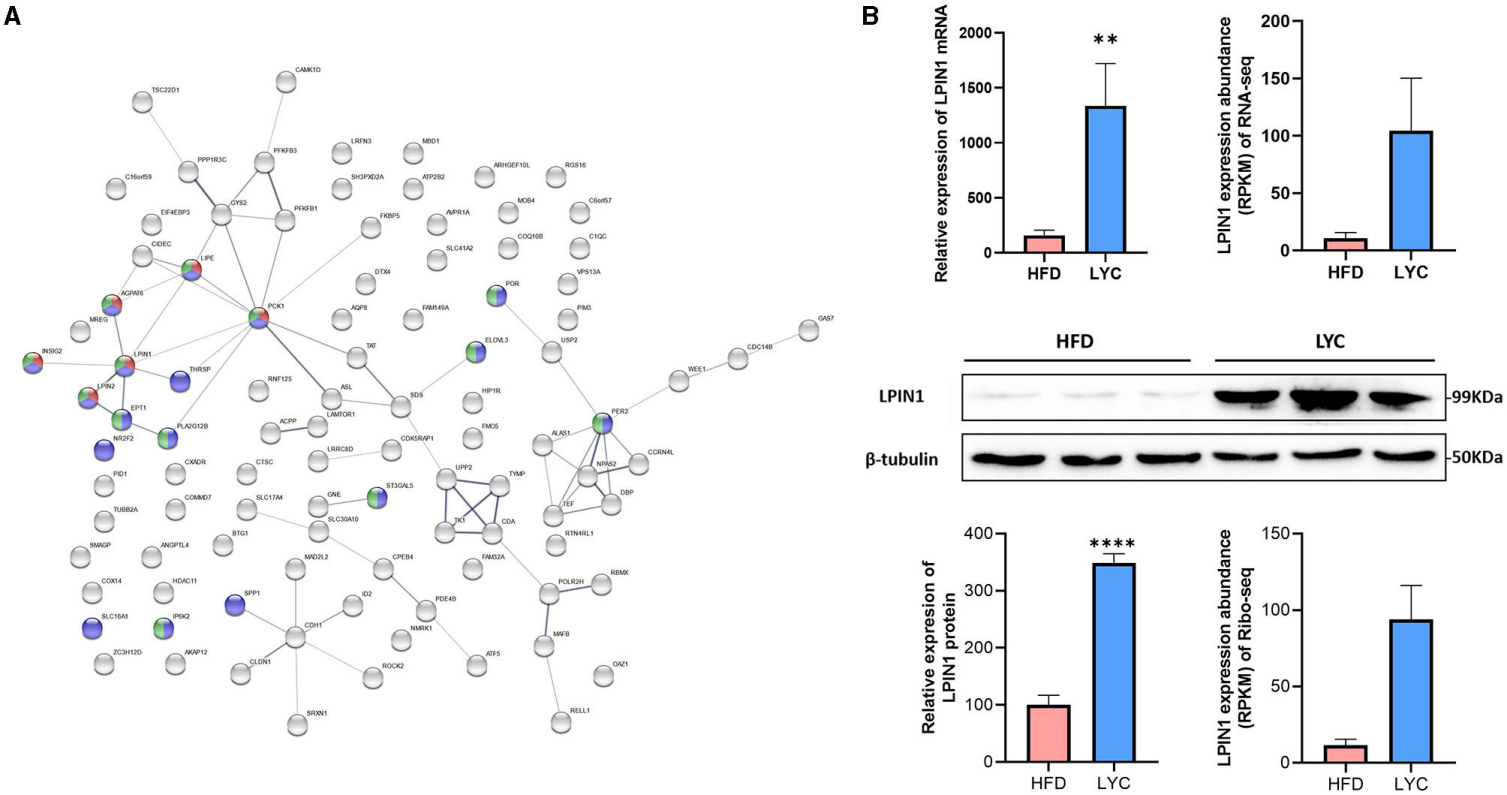
Protein–Protein Interaction Networks. **(A)** Interactive map encoded by differential genes. Nodes represent the proteins. Red: TG metabolic process; Green: lipid metabolic process; purple: cellular lipid metabolic process. **(B)** *LPIN1* expression level base on RT-qPCR, western blot, RNA-seq and Ribo-seq data. **p < 0.01. ****p< 0.0001.

The functional protein association networks were conducted using the online STRING website (https://string-db.org/) and the interactions of the coding proteins of the 112 DEGs (quadrants C and G, Figure 5C) were analyzed to identify the important genes. The results showed *LPIN1* had most crossover nodes in TG metabolic process, lipid metabolic process and cellular lipid metabolic process (Figure 6A). According to real-time fluorescent quantitative PCR, western blot, RNA-seq and Ribo-seq data, compared with the HFD group, *LPIN1* was significantly increased in the LYC group (Figure 6B). *LPIN1* promoted TG metabolism. These results suggest that lycopene may play a lipid-lowering role by regulating the expression of genes related to TG metabolism.

## Discussion

Currently, there is no definitive treatment for NAFLD, and therefore, finding new drugs and treatments is valuable. In our research, liver TG levels were significantly increased in mice fed a HFD (Figure 2A), indicating successful construction of the NAFLD model. In addition, lycopene can improve liver fat accumulation induced by HFD (Figure 2A). This is consistent with previous reports (31–33). *In vitro*, lycopene also significantly alleviated TG accumulation in HepG2 cells induced by OA and PA. Our previous study (20) has shown that different concentrations of lycopene are non-toxic and promoted cell proliferation. In addition, the mice fed with lycopene had no adverse effect, and the body weight increased and the body fat rate decreased significantly. It is suggested that lycopene can be used as a harmless natural dietary supplement.

Translational regulation is a main element of gene expression regulation. Omics measurements proved that translational regulation is obligated to more than half of all regulatory magnitudes (34, 35). Translatome, as a novel technology of omics research in recent years, may provide important information on many biological problems and can detect lncRNA (36, 37), circRNA (38) and pri-miRNA (39), which encode polypeptide. Besides, compared with the transcriptome, the translatome is more able to reflect the changes in expression of the proteome. Schafer et al. reported that in rat liver and heart tissues, ribosomal footprint abundance was better associated with genome-wide protein abundance than RNA-seq data was (40). The low correlation between RNA and protein can be attributed to mRNA half-life, protein folding, degradation and post-translational modification (41, 42). Our study showed a high correlation between mRNA and RPFs abundance (Supplementary Figure 4). Therefore, it is speculated that lycopene may participate in TG metabolism by participating in the regulation of gene transcription and translation.

Firstly, we performed RNA-seq on the livers of mice fed HFD and HFD supplemented with lycopene, and identified 695 upregulated and 432 downregulated DEGs of RNA-seq (Supplementary Figure 3). To narrow the focus of the functional genes, Ribo-seq was performed, and 505 upregulated and 853 downregulated DEGs were identified (Figure 3D). KEGG enrichment analysis of the 1,358 DEGs from Ribo-seq showed that the top 20 pathways included the NAFLD pathway (Figure 4B), suggesting that lycopene plays a role in the improvement of NAFLD by affecting the translational expression of related genes. By combining 1,127 DEGs in transcriptomics with 1,358 DEGs in translatomics, the functional genes of interest were narrowed down to 112 DEGs that regulated in the same direction at transcriptional and translational levels (Figure 5C). Translational responses contribute to the establishment of complex genetic regulation, which cannot be achieved by controlling transcription alone (43).

Protein–protein interaction analysis indicated that lycopene improved hepatic steatosis by regulating the TG metabolic process and lipid metabolic process, mainly regulating the expression of LPIN1 (Figure 6A). LPIN1 can activate mitochondrial fatty acid oxidative metabolism by inducing the expression of the nuclear peroxisome proliferator-activated receptor α (44). In addition, the protein encoded by LPIN1 is involved in the regulation of lipid metabolism in the liver and is associated with the phenotype of fatty liver dystrophy mice (45). Free fatty acids are the major sources of TG stored in the liver. The imbalance of fatty acid absorption and processing is a key factor leading to fat accumulation in the liver (46). Lycopene may play a lipid-lowering role by promoting triglyceride metabolism.

## Conclusions

**Figure 7:**
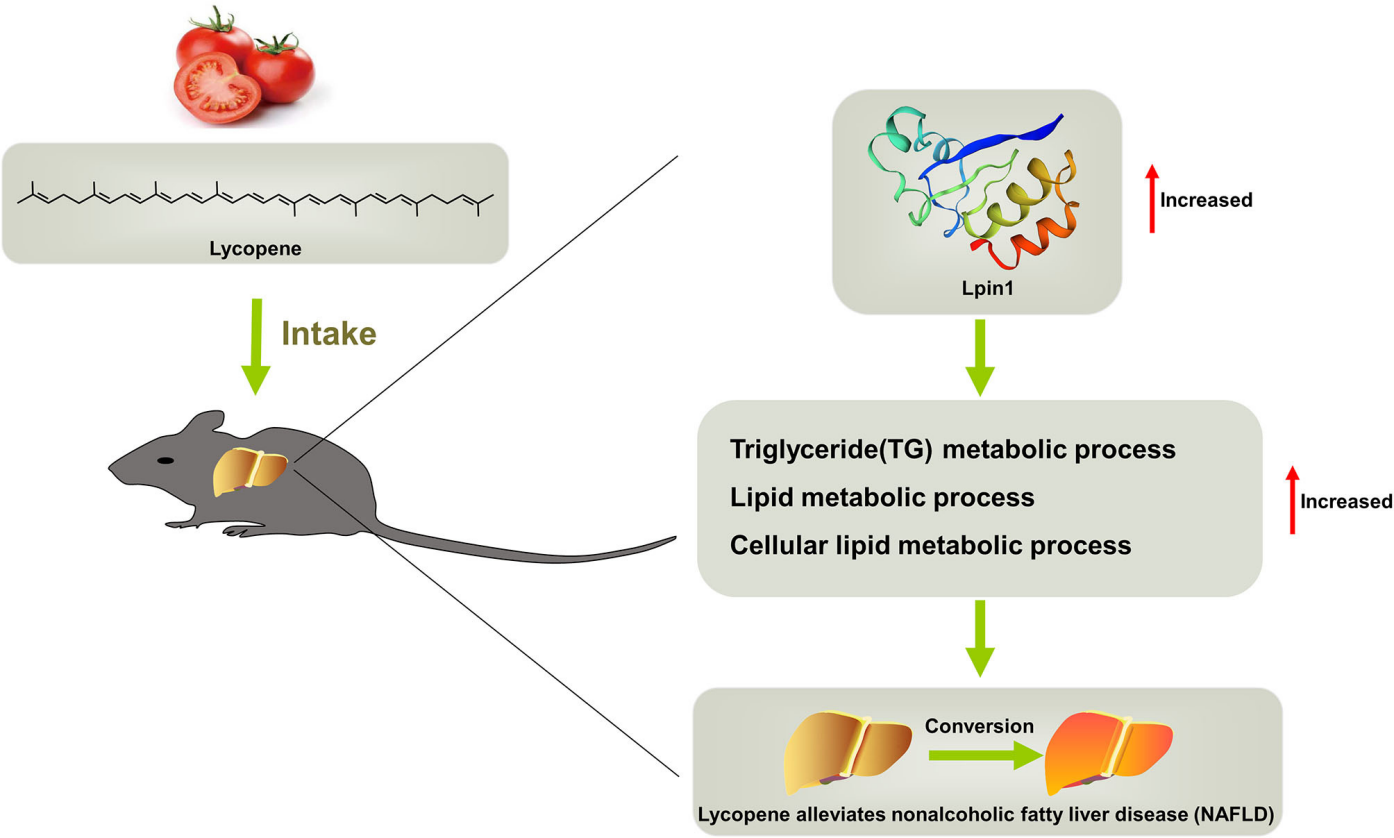
Schematic representation of the mechanisms that mediate lycopene-provided protection against NAFLD.

Based on the results, we concluded that lycopene can effectively alleviate liver steatosis induced by HFD, and can be used as a possible dietary strategy for the control and treatment of NAFLD. This beneficial lipid-lowering effect is due to lycopene increasing the expression of genes related to liver lipid metabolic process at transcriptional and translational levels (Figure 7). Our study fills the gap in the translation profiling of lycopene in the process of alleviating hepatic steatosis, which provides a reasonable molecular regulatory mechanism for this phenotype. However, it has not yet formed an in-depth and systematic regulatory network, which is worth exploring in the next step. In addition, our combined analysis based on transcriptomics and translatomics provides a new way to narrow the range of key functional genes, contributing to a better understanding of the mechanisms involved. This provides a new direction for the further research of the relationship between lycopene and lipid metabolism.

## Data Availability Statement

The datasets presented in this study can be found in online repositories. The names of the repository/repositories and accession number(s) can be found below: www.ncbi.nlm.nih.gov/geo/query/acc.cgi?acc=GSE178322, GSE178322.

## Ethics Statement

The animal study was reviewed and approved by the Committee on the Ethics of Animal Experiments of Guangxi University (No. GXU2019-063).

## Author Contributions

TH, JY, LZ, and GX conceived the project and design the protocol. ZM, SL, ZL, WM, and LY performed the experiments. TH, KL, DY, ZS, and YL performed the data analysis. TH, QF, LZ, and GX wrote the manuscript. All authors contributed to the article and approved the submitted version.

## Funding

This work was supported by grants from the National Key R&D Program of China (2018YFD0500402), Guangxi Science Foundation for Distinguished Young Scholars (2020GXNSFFA297008), Guangxi Science and Technology Base and Talents Project (AD18281085), Guangxi Natural Science Foundation (2019GXNSFDA245029), Scientific Research and Technology Development Major Project of Nanning (20192004-1), Scientific Research and Technological Development Program of Yongning District of Nanning City (20180101B), State Key Laboratory for Conservation and Utilization of Subtropical Agro-bioresources (SKLCUSA-a202006), and Training Project of High-level Professional and Technical Talents of Guangxi University.

## Conflict of Interest

The authors declare that the research was conducted in the absence of any commercial or financial relationships that could be construed as a potential conflict of interest.

## Publisher's Note

All claims expressed in this article are solely those of the authors and do not necessarily represent those of their affiliated organizations, or those of the publisher, the editors and the reviewers. Any product that may be evaluated in this article, or claim that may be made by its manufacturer, is not guaranteed or endorsed by the publisher.

## Supplementary Material

Supplementary Figure 1 KEGG pathway diagram, **(A)** oxidative phosphorylation pathway. **(B)** Non-alcoholic fatty liver disease pathway. Red represents DEGs.

Supplementary Figure 2 Principal component analysis (PCA) in RNA-seq.

Supplementary Figure 3 Volcano plots of DEGs in RNA-seq, |log_2_ fold change|>1 and *p* < 0.01.

Supplementary Figure 4 The scatter plot of correlation between mRNA abundance and RPF abundance. Red: HFD; blue: LYC.

Supplementary Table 1 Nutritional composition of mice feed.

Supplementary Table 2 Differentially expressed genes in Ribo-seq.

Supplementary Table 3 GO biological process of DEGs in Ribo-seq.

Supplementary Table 4 KEGG pathways of DEGs in Ribo-seq.

Supplementary Table 5 Differentially expressed genes in RNA-seq.

Supplementary Table 6 The gene information in different quadrants.

## Abbreviations

HFD: high fat diet
LYC: high-fat feed containing lycopene
DEGs: differentially expressed genes
VLDLs: very low-density lipoproteins
NAFLD: non-alcoholic fatty liver disease
RNA-seq: RNA sequencing
Ribo-seq: ribosome footprint sequencing
OAPA: oleic and palmitic acids
TG: triglyceride
qPCR: quantitative polymerase chain reaction
RFPs: ribosome footprints
RPF: ibosome protected fragment
RPKM: reads per kilobase per million reads
GO: gene ontology
BP: biological process
CC: cellular component
MF: molecular function
KEGG: Kyoto Encyclopedia of Genes and Genomes.

## References

[1] Nguyen P Leray V Diez M Serisier S Le Bloc'H J Siliart B. Liver lipid metabolism. J Anim Physiol Anim Nutr. (2008) 92:272–83. 10.1111/j.1439-0396.2007.00752.x 18477307

[2] Jian T Yu C Ding X Chen J Li J Zuo Y. Hepatoprotective effect of seed coat ofeuryale ferox extract in non-alcoholic fatty liver disease induced by high-fat diet in mice by increasing IRs-1 and inhibiting CYP2E1. J Oleo Sci. (2019) 68:581–9. 10.5650/jos.ess19018 31092797

[3] Vernon G Baranova A Younossi ZM. Systematic review: the epidemiology and natural history of non-alcoholic fatty liver disease and non-alcoholic steatohepatitis in adults. Aliment Pharmacol Ther. (2011) 34:274–85. 10.1111/j.1365-2036.2011.04724.x 21623852

[4] Komine S Akiyama K Warabi E Oh S Kuga K Ishige K. Exercise training enhances *in vivo* clearance of endotoxin and attenuates inflammatory responses by potentiating Kupffer cell phagocytosis. Sci Rep. (2017) 7:11977. 10.1038/s41598-017-12358-8 28931917 PMC5607327

[5] Moore MP Cunningham RP Dashek RJ Mucinski JM Rector RS. A Fad too Far? Dietary strategies for the prevention and treatment of NAFLD. Obesity. (2020) 28:1843–52. 10.1002/oby.22964 32893456 PMC7511422

[6] Grabowska M Wawrzyniak D Rolle K Chomczyński P Oziewicz S Jurga S. Let food be your medicine: nutraceutical properties of lycopene. Food Funct. (2019) 10:3090–102. 10.1039/C9FO00580C 31120074

[7] Li N Wu X Zhuang W Xia L Chen Y Wu C. Tomato and lycopene and multiple health outcomes: umbrella review. Food Chem. (2021) 343:128396. 10.1016/j.foodchem.2020.128396 33131949

[8] Kawata A Murakami Y Suzuki S Fujisawa S. Anti-inflammatory activity of β-carotene, lycopene and Tri-n-butylborane, a scavenger of reactive oxygen species. In vivo. (2018) 32:255–64. 10.21873/invivo.11232 29475907 PMC5905192

[9] Zeng Z He W Jia Z Hao S. Lycopene improves insulin sensitivity through inhibition of STAT3/Srebp-1c-mediated lipid accumulation and inflammation in mice fed a high-fat diet. Exp Clin Endocrinol Diabetes. (2017) 125:610–7. 10.1055/s-0043-101919 28472825

[10] Jhou BY Song TY Lee I Hu ML Yang NC. Lycopene inhibits metastasis of human liver adenocarcinoma SK-Hep-1 cells by downregulation of NADPH oxidase 4 protein expression. J Agric Food Chem. (2017) 65:6893–903. 10.1021/acs.jafc.7b03036 28723216

[11] Lindshield BL Canene-Adams K Erdman JJ. Lycopenoids: are lycopene metabolites bioactive? Arch Biochem Biophys. (2007) 458:136–40. 10.1016/j.abb.2006.09.012 17067545

[12] Ahn J Lee H Jung CH Ha T. Lycopene inhibits hepatic steatosis via microRNA-21-induced downregulation of fatty acid-binding protein 7 in mice fed a high-fat diet. Mol Nutr Food Res. (2012) 56:1665–74. 10.1002/mnfr.201200182 22968990

[13] Chung J Koo K Lian F Hu KQ Ernst H Wang XD. Apo-10'-lycopenoic acid, a lycopene metabolite, increases sirtuin 1 mRNA and protein levels and decreases hepatic fat accumulation in ob/ob mice. J Nutr. (2012) 142:405–10. 10.3945/jn.111.150052 22259190 PMC3278264

[14] Elvira-Torales LI Navarro-González I González-Barrio R Martín-Pozuelo G Doménech G Seva J. Tomato juice supplementation influences the gene expression related to steatosis in rats. Nutrients. (2018) 10:1215. 10.3390/nu10091215 30200543 PMC6165399

[15] Sousa MJ Liu X Oke A Arora R Franciosi F Viville S. DAZL and CPEB1 regulate mRNA translation synergistically during oocyte maturation. J Cell Sci. (2016) 129:1271–82. 10.1242/jcs.179218 26826184 PMC4813292

[16] Miettinen TP Kang JH Yang LF Manalis SR. Mammalian cell growth dynamics in mitosis. Elife. (2019) 8:e44700. 10.7554/eLife.44700 31063131 PMC6534395

[17] Fujii K Shi Z Zhulyn O Denans N Barna M. Pervasive translational regulation of the cell signalling circuitry underlies mammalian development. Nat Commun. (2017) 8:14443. 10.1038/ncomms14443 28195124 PMC5316868

[18] Liu M Ge R Liu W Liu Q Xia X Lai M. Differential proteomics profiling identifies LDPs and biological functions in high-fat diet-induced fatty livers. J Lipid Res. (2017) 58:681–94. 10.1194/jlr.M071407 28179399 PMC5392744

[19] Lu Y Shao M Xiang H Zheng P Wu T Ji G. Integrative transcriptomics and metabolomics explore the mechanism of kaempferol on improving nonalcoholic steatohepatitis. Food Funct. (2020) 11:10058–69. 10.1039/D0FO02123G 33135718

[20] Gygi SP Rochon Y Franza BR Aebersold R. Correlation between protein and mRNA abundance in yeast. Mol Cell Biol. (1999) 19:1720–30. 10.1128/MCB.19.3.1720 10022859 PMC83965

[21] Maier T Güell M Serrano L. Correlation of mRNA and protein in complex biological samples. FEBS Lett. (2009) 583:3966–73. 10.1016/j.febslet.2009.10.036 19850042

[22] Ingolia NT Ghaemmaghami S Newman JR Weissman JS. Genome-wide analysis *in vivo* of translation with nucleotide resolution using ribosome profiling. Science. (2009) 324:218–23. 10.1126/science.1168978 19213877 PMC2746483

[23] Liu S Yang D Yu L Aluo Z Zhang Z Qi Y. Effects of lycopene on skeletal muscle-fiber type and high-fat diet-induced oxidative stress. J Nutr Biochem. (2021) 87:108523. 10.1016/j.jnutbio.2020.108523 33039582

[24] Zhao B Liu H Wang J Liu P Tan X Ren B. Lycopene supplementation attenuates oxidative stress, neuroinflammation, and cognitive impairment in aged CD-1 mice. J Agric Food Chem. (2018) 66:3127–36. 10.1021/acs.jafc.7b05770 29509007

[25] Qi Y Zhang Z Liu S Aluo Z Zhang L Yu L. Zinc supplementation alleviates lipid and glucose metabolic disorders induced by a high-fat diet. J Agric Food Chem. (2020) 68:5189–200. 10.1021/acs.jafc.0c01103 32290656

[26] Lian X Guo J Gu W Cui Y Zhong J Jin J. Genome-wide and experimental resolution of relative translation elongation speed at individual gene level in human cells. PLoS Genet. (2016) 12:e1005901. 10.1371/journal.pgen.1005901 26926465 PMC4771717

[27] Xiao CL Mai ZB Lian XL Zhong JY Jin JJ He QY. FANSe2: a robust and cost-efficient alignment tool for quantitative next-generation sequencing applications. PLoS ONE. (2014) 9:e94250. 10.1371/journal.pone.0094250 24743329 PMC3990525

[28] Huang T Yu J Luo Z Yu L Liu S Wang P. Translatome analysis reveals the regulatory role of betaine in high fat diet (HFD)-induced hepatic steatosis. Biochem Bioph Res Commun. (2021) 575:20–7. 10.1016/j.bbrc.2021.08.058 34454176

[29] Mortazavi A Williams BA McCue K Schaeffer L Wold B. Mapping and quantifying mammalian transcriptomes by RNA-Seq. Nat Methods. (2008) 5:621–8. 10.1038/nmeth.1226 18516045 PMC13303166

[30] Robinson MD McCarthy DJ Smyth GK. edgeR: a Bioconductor package for differential expression analysis of digital gene expression data. Bioinformatics. (2010) 26:139–40. 10.1093/bioinformatics/btp616 19910308 PMC2796818

[31] Wan X Yang Z Ji H Li N Yang Z Xu L. Effects of lycopene on abdominal fat deposition, serum lipids levels and hepatic lipid metabolism-related enzymes in broiler chickens. Anim Biosci. (2021) 34:385–92. 10.5713/ajas.20.0432 33152222 PMC7961199

[32] Ota T. Prevention of NAFLD/NASH by Astaxanthin and β-Cryptoxanthin. Adv Exp Med Biol. (2021) 1261:231–8. 10.1007/978-981-15-7360-6_21 33783746

[33] Lee Y Hu S Park YK Lee JY. Health benefits of carotenoids: a role of carotenoids in the prevention of non-alcoholic fatty liver disease. Prev Nutr Food Sci. (2019) 24:103–13. 10.3746/pnf.2019.24.2.103 31328113 PMC6615349

[34] Zhao J Qin B Nikolay R Spahn C Zhang G. Translatomics: the global view of translation. Int J Mol Sci. (2019) 20:212. 10.3390/ijms20010212 30626072 PMC6337585

[35] Ingolia NT Brar GA Rouskin S McGeachy AM Weissman JS. The ribosome profiling strategy for monitoring translation *in vivo* by deep sequencing of ribosome-protected mRNA fragments. Nat Protoc. (2012) 7:1534–50. 10.1038/nprot.2012.086 22836135 PMC3535016

[36] Kondo T Plaza S Zanet J Benrabah E Valenti P Hashimoto Y. Small peptides switch the transcriptional activity of Shavenbaby during Drosophila embryogenesis. Science. (2010) 329:336–9. 10.1126/science.1188158 20647469

[37] Anderson DM Anderson KM Chang CL Makarewich CA Nelson BR McAnally JR. A micropeptide encoded by a putative long noncoding RNA regulates muscle performance. Cell. (2015) 160:595–606. 10.1016/j.cell.2015.01.009 25640239 PMC4356254

[38] Zhang M Huang N Yang X Luo J Yan S Xiao F. A novel protein encoded by the circular form of the SHPRH gene suppresses glioma tumorigenesis. Oncogene. (2018) 37:1805–14. 10.1038/s41388-017-0019-9 29343848

[39] Lauressergues D Couzigou JM Clemente HS Martinez Y Dunand C Bécard G. Primary transcripts of microRNAs encode regulatory peptides. Nature. (2015) 520:90–3. 10.1038/nature14346 25807486

[40] Schafer S Adami E Heinig M Rodrigues K Kreuchwig F Silhavy J. Translational regulation shapes the molecular landscape of complex disease phenotypes. Nat Commun. (2015) 6:7200. 10.1038/ncomms8200 26007203 PMC4455061

[41] Schwanhäusser B Busse D Li N Dittmar G Schuchhardt J Wolf J. Global quantification of mammalian gene expression control. Nature. (2011) 473:337–42. 10.1038/nature10098 21593866

[42] Nedialkova DD Leidel SA. Optimization of codon translation rates via tRNA modifications maintains proteome integrity. Cell. (2015) 161:1606–18. 10.1016/j.cell.2015.05.022 26052047 PMC4503807

[43] Loya CM Van Vactor D Fulga TA. Understanding neuronal connectivity through the post-transcriptional toolkit. Genes Dev. (2010) 24:625–35. 10.1101/gad.1907710 20360381 PMC2849119

[44] Finck BN Gropler MC Chen Z Leone TC Croce MA Harris TE. Lipin 1 is an inducible amplifier of the hepatic PGC-1alpha/PPARalpha regulatory pathway. Cell Metab. (2006) 4:199–210. 10.1016/j.cmet.2006.08.005 16950137

[45] Péterfy M Phan J Xu P Reue K. Lipodystrophy in the fld mouse results from mutation of a new gene encoding a nuclear protein, lipin. Nat Genet. (2001) 27:121–4. 10.1038/83685 11138012

[46] Musso G Gambino R Cassader M. Recent insights into hepatic lipid metabolism in non-alcoholic fatty liver disease (NAFLD). Prog Lipid Res. (2009) 48:1–26. 10.1016/j.plipres.2008.08.001 18824034

