# Supplementary figures and images for: Translatomics Probes Into the Role of Lycopene on Improving Hepatic Steatosis Induced by High-Fat Diet
Source: Front Nutr. 2021 Nov 2;8:727785. doi: 10.3389/fnut.2021.727785 (PMC8594419; doi:10.3389/fnut.2021.727785)

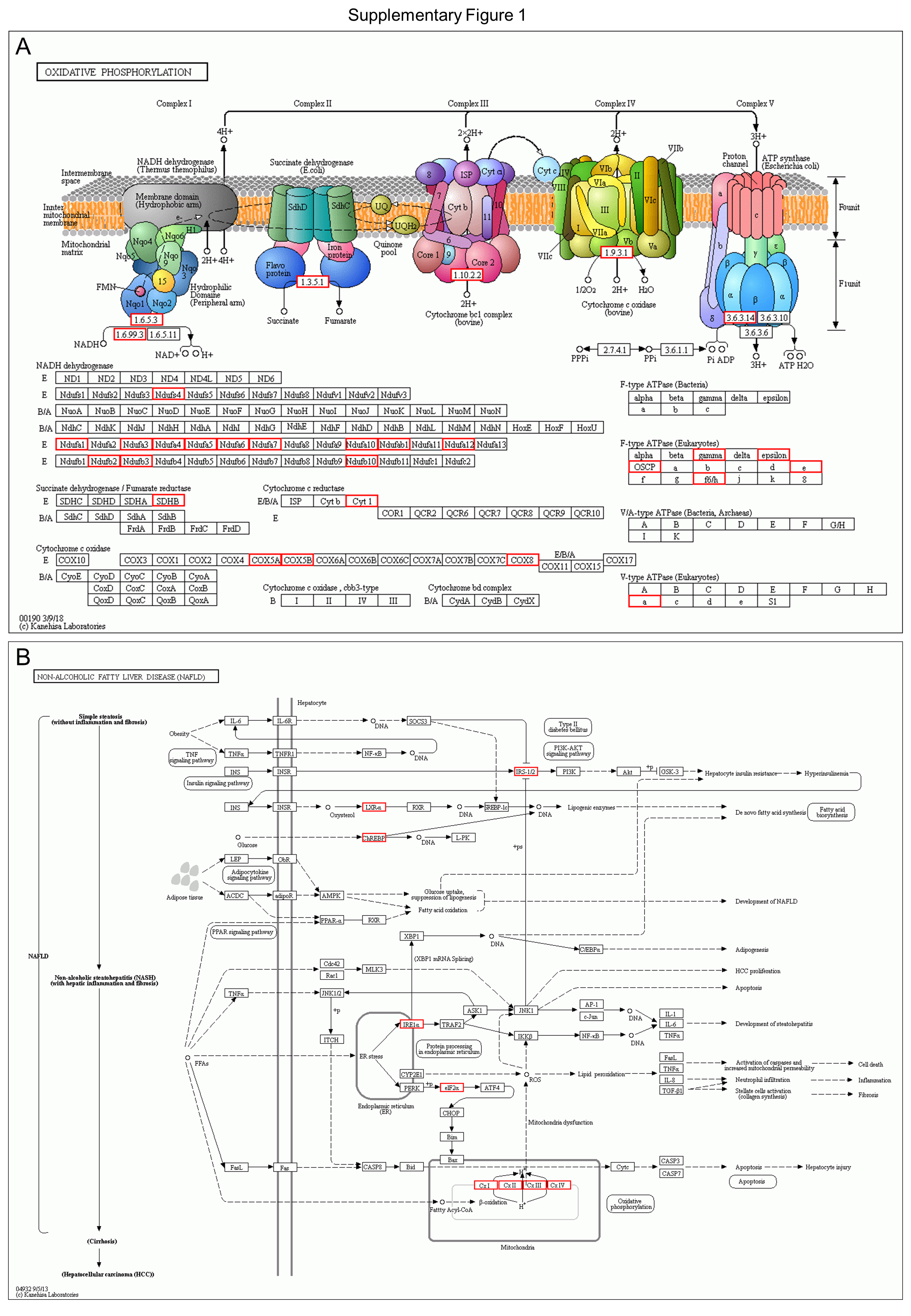

Supplement: Supplementary Figure 1 — KEGG pathway diagram, (A) oxidative phosphorylation pathway. (B) Non-alcoholic fatty liver disease pathway. Red represents DEGs. [file Image_1.TIF]

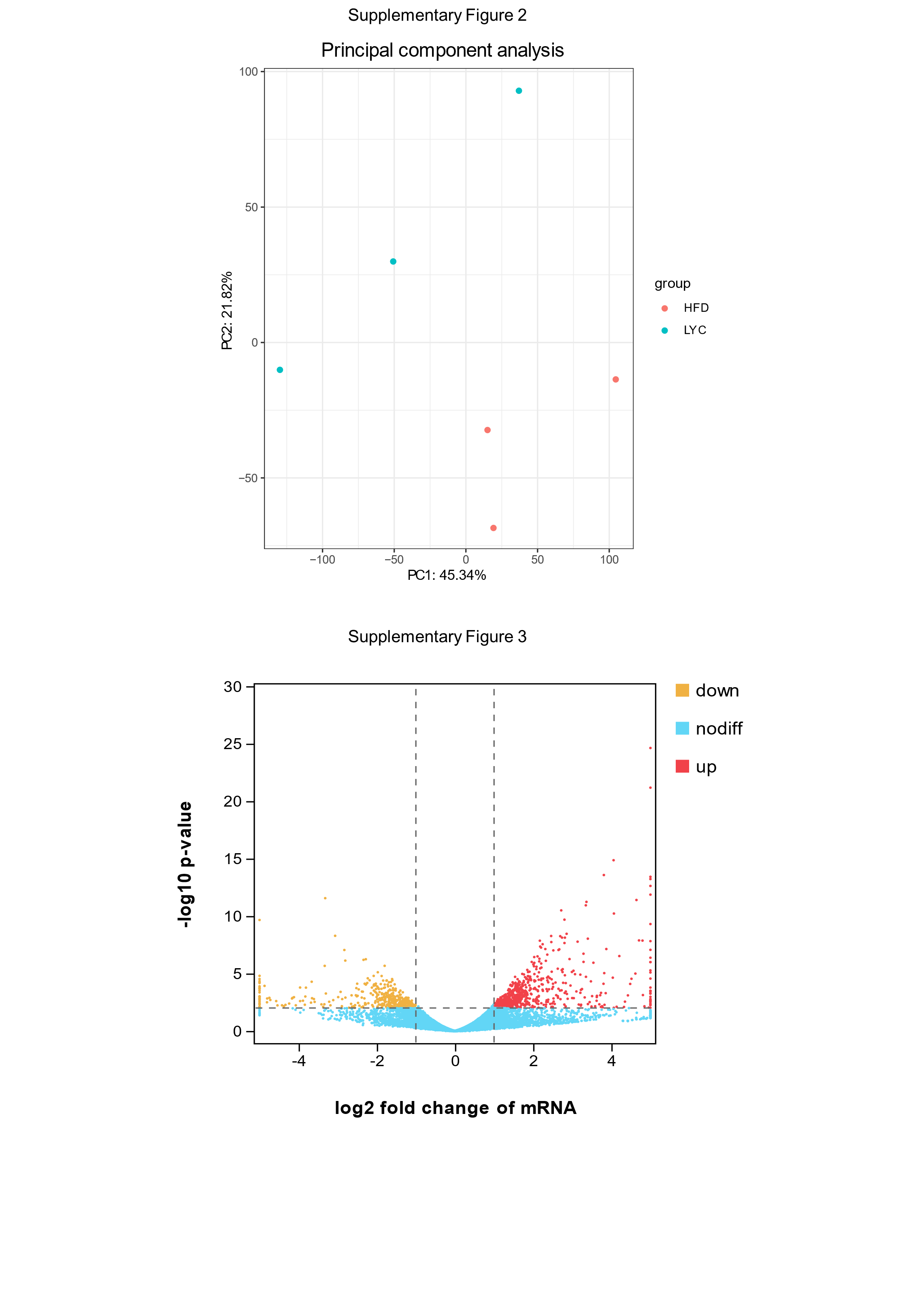

Supplement: Supplementary Figure 2 — Principal component analysis (PCA) in RNA-seq. [file Image_2.TIF]

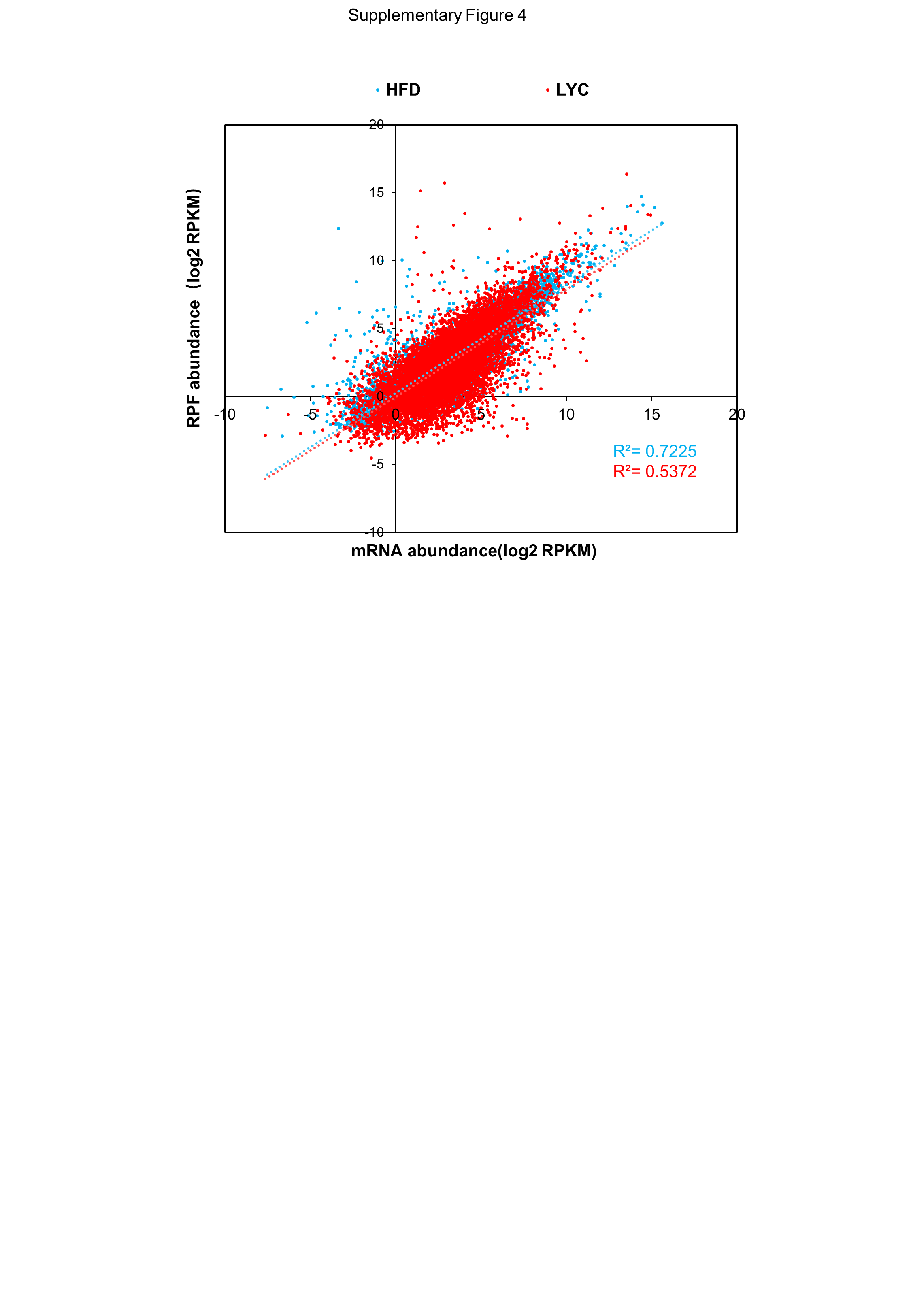

Supplement: Supplementary Figure 3 — Volcano plots of DEGs in RNA-seq, |log2 fold change|>1 and p < 0.01. [file Image_3.TIF]

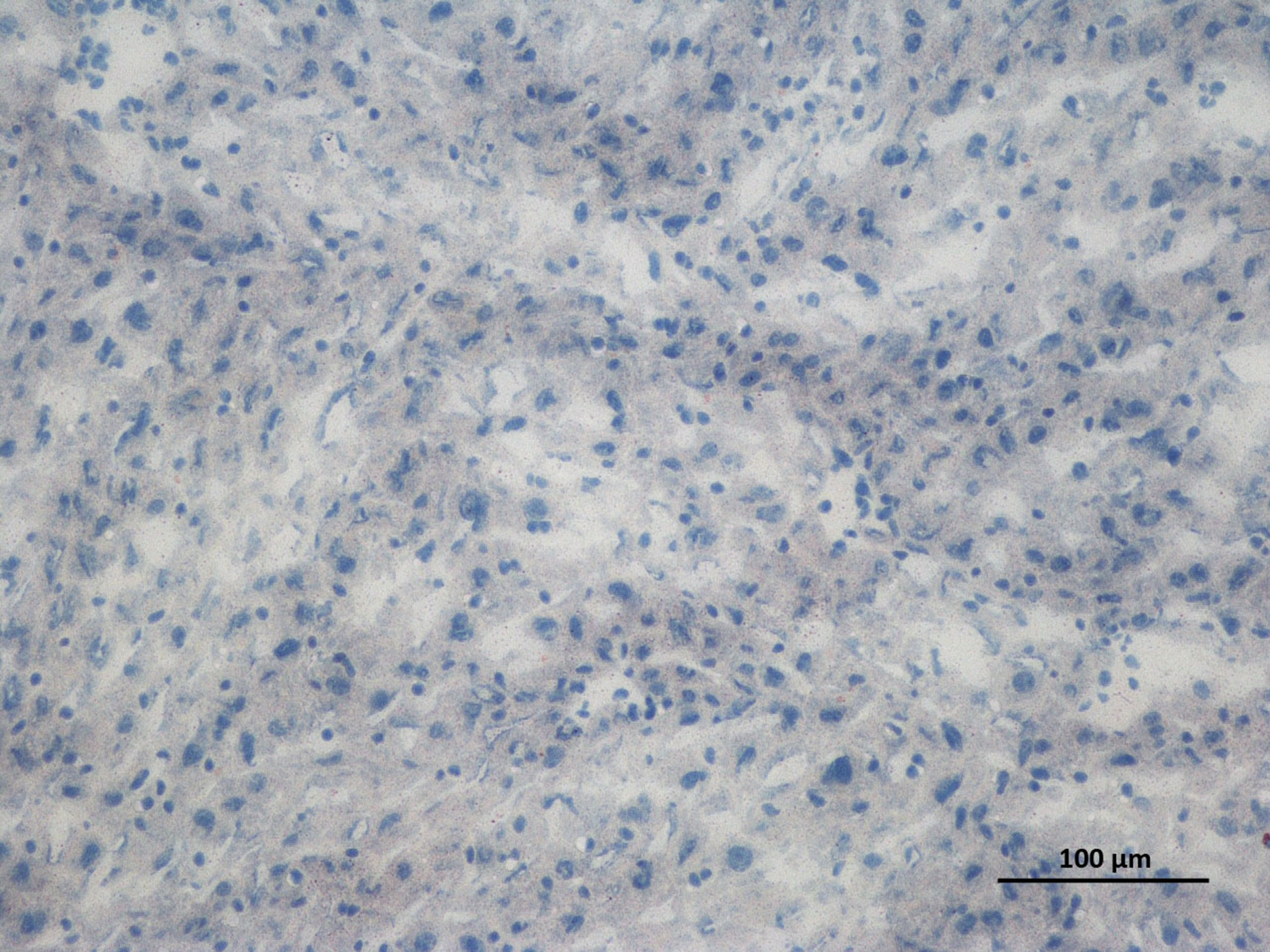

Supplement: Supplementary Figure 4 — The scatter plot of correlation between mRNA abundance and RPF abundance. Red: HFD; blue: LYC. [file Image_4.JPEG]

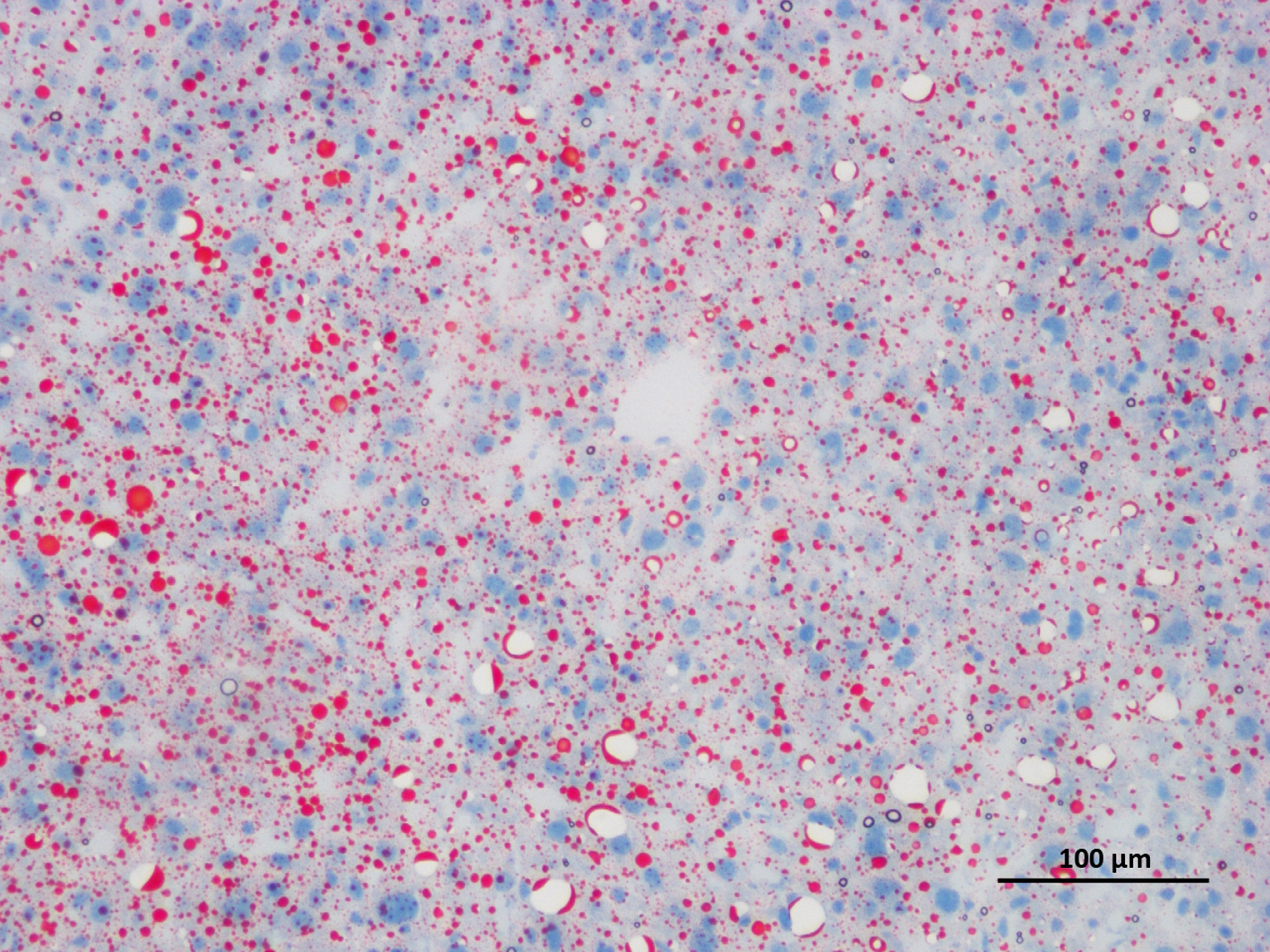

Supplement: Supplementary file 11 [file Image_5.JPEG]

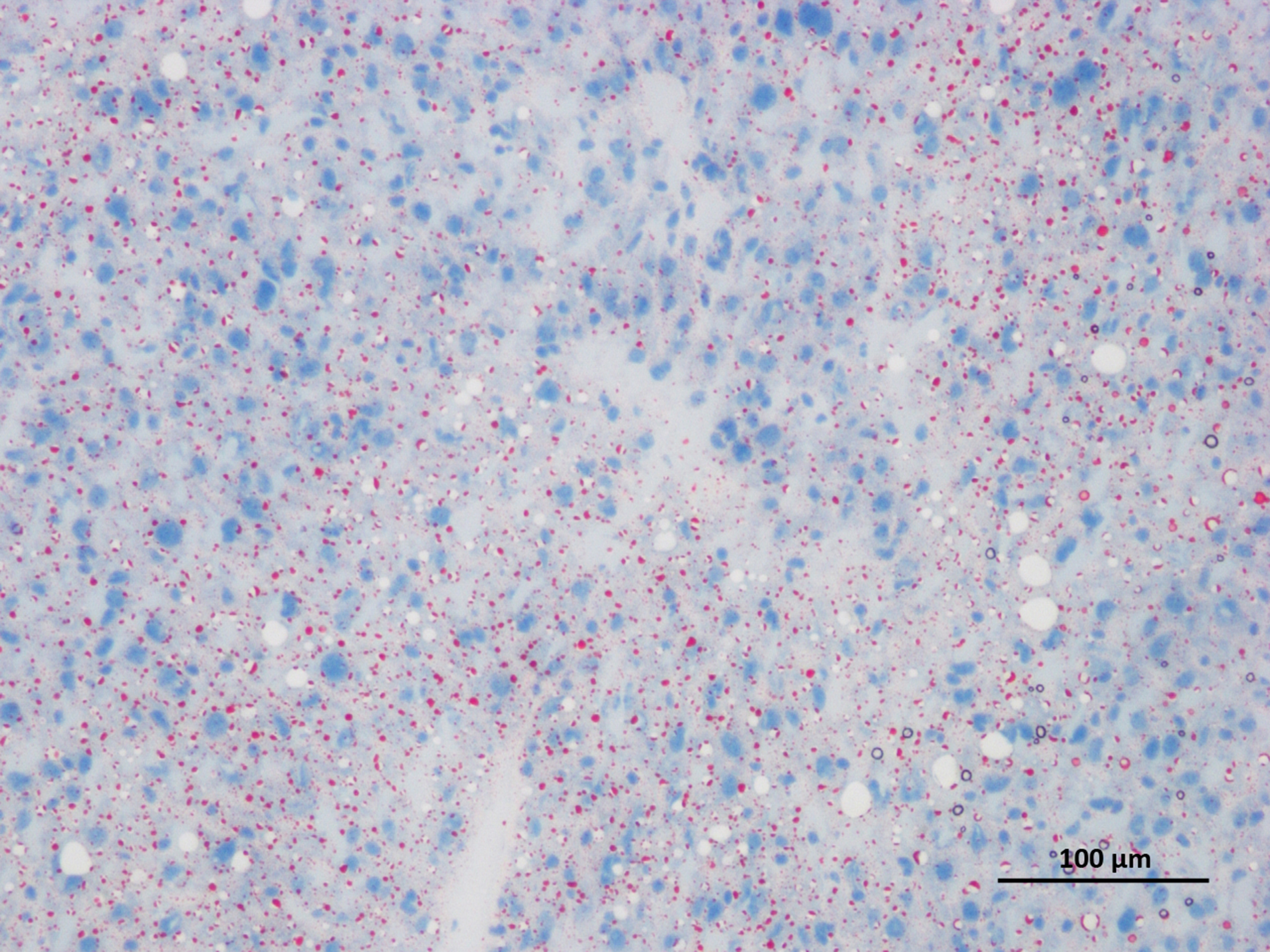

Supplement: Supplementary file 12 [file Image_6.JPEG]
