# Supplementary material for: Translatomics Probes Into the Role of Lycopene on Improving Hepatic Steatosis Induced by High-Fat Diet
Source: Front Nutr. 2021 Nov 2;8:727785. doi: 10.3389/fnut.2021.727785 (PMC8594419; doi:10.3389/fnut.2021.727785)
Supplement: Supplementary Table 1 — Nutritional composition of mice feed. [file Table_1.DOCX]

Supplementary Table 1: Nutritional composition of mice feed

| Nutrient, % | CK group | HFD and LYC groups |
| --- | --- | --- |
| protein | 19.2 | 24 |
| carbohydrates | 67.3 | 41 |
| fats | 4.3 | 24 |
